# Study on the Decoupled Transfer of Heat and Mass in Wire Variable Polarity Plasma Arc Welding

**DOI:** 10.3390/ma13051073

**Published:** 2020-02-28

**Authors:** Fan Jiang, Cheng Li, Bin Xu, Shinichi Tashiro, Manabu Tanaka, Shujun Chen

**Affiliations:** 1 Engineering Research Center of Advanced Manufacturing Technology for Automotive Components, Ministry of Education, Beijing University of Technology, Beijing 100124, China; jiangfan@bjut.edu.cn (F.J.); licheng_bjut@163.com (C.L.); sjchen@bjut.edu.cn (S.C.); 2 Joining and Welding Research Institute, Osaka University, Osaka 5670047, Japan; tashiro@jwri.osaka-u.ac.jp (S.T.); tanaka@jwri.osaka-u.ac.jp (M.T.); 3 School of Materials Science and Engineering, Shandong Jianzhu University, Jinan 250101, China

**Keywords:** decoupling control, heat and mass transfer, VPPA/PMIG, aluminum alloy

## Abstract

A hybrid arc-wire welding method based on the variable polarity plasma arc (VPPA) and variable polarity pulse metal inert-gas (VP-PMIG) was proposed for manufacturing aluminum alloys. This paper aims to clarify the decoupling control process of heat and mass transfer in the hybrid welding process. To understand the arc physics and analyze the mass transfer behavior, the hybrid arc shape and droplet cross-sectional area with different parameters were obtained by high speed video photography. Further, the melting area of the base metal was analyzed by macro-metallography of the weld bead cross-section to study the heat transfer. It is found that the hybrid arc shape changes with time. The VPPA main arc is deflected to one side by the VP-PMIG, making the temperature distribution asymmetric, and during the VP-PMIG pulse necking occurs. The cross-sectional area of the droplet is more obviously affected by the VP-PMIG current than the VPPA current. Meanwhile, the VPPA current dominates the melting area of the base metal. Therefore, we conclude that heat transfer to the base metal is from the VPPA, while droplet transfer is mainly controlled by the VP-PMIG arc. These conclusions are confirmed by analyzing the decoupling degree of heat and mass transfer of the base metal by the VPPA and VP-PMIG arc.

## 1. Introduction

Within industrial development, aluminum alloys are widely used in aerospace, machinery manufacturing and other fields [1]. At present, the arc welding process is one of the most widely used manufacturing methods for aluminum alloys [2]. Due to the special material properties of aluminum alloy, the heat and mass transfer of aluminum alloy welding is very important [3]. Therefore, the heat and mass transfer in the welding process of aluminum alloy has always been a research hotspot. Meanwhile, some researchers use some special methods to study the heat and mass transfer in welding process of steel. In order to qualitatively analyze the heat transfer in double electrode micro-plasma arc welding process, Li et al. [4] obtained the thermal cycle curve of base metal in the welding process by thermocouple. Recently, Jiang et al. [5] designed a set of devices which can measure the instantaneous heat transfer of the variable polarity plasma arc (VPPA) to the base metal. Additionally, the heat transfer is indirectly calculated by the temperature difference of the cooling water. Pomare M et al. [6] analyzed droplet melting analysis by using synchronized high-speed video, and high-speed data acquisition was used to evaluate arc behavior and melting rate. It considered that the melting rate of the EP phase in which the tungsten electrode is connected to positive pole of welding power supply, was related to the prepulse droplet volume. Hence, it provided an effective method for studying the mass transfer in the welding process. 

However, the mass transfer of single arc welding is limited by the welding current, so mass transfer efficiency of a single arc is too low. Based on the traditional arc welding process, a series of hybrid arc welding methods are proposed to combine the advantages of different kinds of arcs, which can improve the mass transfer efficiency. By combining the two arcs, tandem tungsten inert gas (TIG) welding [7,8] and tandem gas metal arc welding (GMAW) [9,10] have realized high welding efficiency. The complementary advantages of different heat sources can also be realized by combining two or more heat sources, such as TIG-MIG (tungsten inert gas-metal inert gas) welding [11,12,13], plasma-MIG hybrid welding [14,15] and hybrid laser-arc welding [16,17], etc. In these welding methods, the base metal is a coupling carrier, which couples heat and mass transfer. High current weld quality is difficult to control because of this coupling [18]. 

In order to realize the decoupling control of the heat and mass transfer, Zhang et al. [19] proposed the bypass coupled GMAW method. The bypass TIG arc is established between the wire and a tungsten electrode. So, it is possible to control the base metal current while increasing the deposition rate. Zhang et al. [20] subsequently developed arcing-wire gas tungsten arc welding (GTAW), another method that can decouple the heat and mass transfer. In this process, a TIG arc called the main arc occurs between the tungsten electrode and the base metal, and a melt inert-gas welding (MIG) arc called the bypass arc is established between the tungsten electrode and the wire. According to this principle, Chen et al. [21] proposed arcing-wire PAW, which combines the advantages of PAW and GMAW. Ma et al. [22] developed tri-arc double wire welding, which can improve the efficiency and decouple the heat and mass transfer. In this method, a variable polarity arc can be established between two wires by loading a variable polarity welding supply to a tandem GMAW system. Lu et al. [23] proposed cross-coupling arc welding, which can decouple the heat and mass transfer by cross coupling a GTAW arc and GMAW arc. In this process, one arc is formed between the tungsten electrode and base metal, while the other one is formed between the two wires. However, none of these welding methods are completely aimed at aluminum alloy welding. 

Based on the above research, VPPA/PMIG welding was proposed, which is a deep decoupling control welding method for aluminum alloy with two independent variable polarity power sources. In this process, a variable polarity pulse metal inert-gas (VP-PMIG) arc is added to a conventional VPPA system. It is more effective for a droplet to fall by adding bypass pulse current. However, the characteristics of the hybrid arc, droplet transfer, and the degree of decoupling of heat and mass of this new method are not clearly understood. In particular, the decoupling degree is the key to realizing the decoupling control of heat and mass transfer. Therefore, the study focuses on the decoupling degree of heat and mass transfer in the new welding method.

Here, we built an experimental platform for arcing-wire VPPA welding to characterize the system. The manuscript is divided into three sections. In the first section, the characteristics of hybrid arc and droplet transfer were analyzed by using a high-speed video camera to dynamically observe the shape of the hybrid arc and droplet cross-sectional area. In the second section, the influence of the hybrid arc currents, including VPPA and VP-PMIG currents on the mass transfer, was studied by contrastively analyzing the influence of each current on the growth rate of the droplet cross-sectional area. Meanwhile, the degree of decoupling control on mass transfer was calculated according to the changing rate of the droplet cross-sectional area change for each current. In the third section, the metallographic microstructure of corresponding weld cross-sections were studied to determine the influence of hybrid arc currents on heat transfer. The degree of decoupling control on heat transfer was calculated according to the changing rate of the melting area of the base metal with each current. This research establishes a theoretical foundation for the application and optimization of the arcing-wire VPPA welding process.

## 2. Experimental Procedure

### 2.1. Experimental Materials

The aluminum alloy 5A06, with a thickness of 6 mm, was adopted as the base metal, having the chemical composition shown in Table 1. The aluminum alloy 5A06 fortification state is H112 state of the aluminum and aluminum alloy hot extrusion profile. The welding wire ER4043, with a diameter of 1.2 mm, having the chemical composition shown in Table 2, was selected as the filling material. Pure argon was used as both the plasma and shielding gas. 

### 2.2. Experimental System and Principle

The experimental system mainly includes welding and waveform coupling systems. The welding system consists of a VPPA welding power source developed by Beijing University of Technology, a CLOOS (Carl Cloos Schweißtechnik GmbH) MIG welding power source, and a wire feeder. A high-speed camera (IDT Y4 series) was used to record the droplet transfer process in real time at a frame rate of 2000 fps. A water tank was used for cooling the plasma torch. The wire feeder provides the wire for the hybrid arc. The waveform coupling system is used to couple the voltage signal of the hybrid arc. A robot is used to fix the plasma torch and MIG torch. The base metal is fixed on the platform with directional movement for welding.

**Figure 1:**
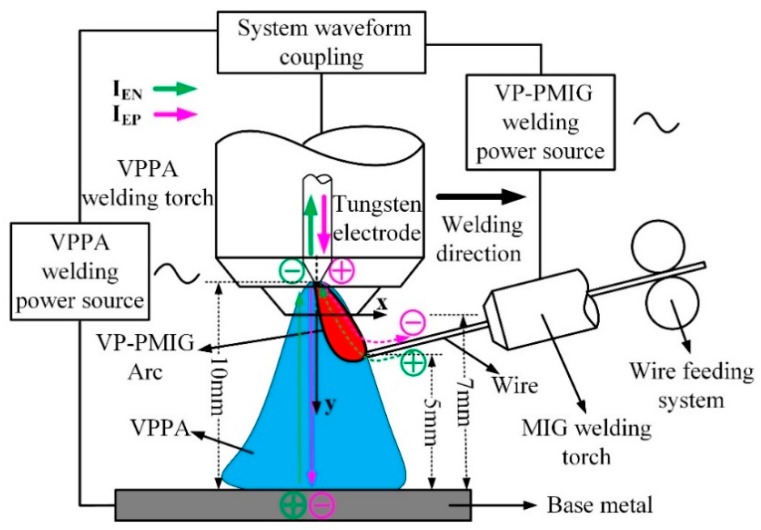
Schematic diagram of the coupling between the variable polarity plasma arc (VPPA) and variable polarity pulse metal inert-gas (VP-PMIG) arc.

Figure 1 is a schematic diagram of the coupling between the VPPA and VP-PMIG arc. In Figure 1, the VPPA, as the main arc, is established between the tungsten electrode and base metal. The VP-PMIG arc, as the bypass arc, is established between the common tungsten electrode and the wire. The two arcs are controlled by two welding machine, separately. We can adjust the parameters of one of them without any effect on the other. Additionally, the heat input is dominated by VPPA, while the VP-PMIG arc has little influence. That means the current of VPPA can be adjusted for satisfying the necessary heat input, without effect on the droplet transfer behavior. The VP-PMIG arc dominates the droplet transfer behavior. Therefore, the heat input can be adjusted in a large range by VPPA for aluminum alloy manufacturing with the high deposition for high welding efficiency and high quality. In order to ensure the stability of the welding process, the polarity of the tungsten electrode needs to match the wire polarity. This is achieved by the VPPA current being switched synchronously with the VP-PMIG current. The distance between the tungsten electrode and the base metal is 10 mm. The distance between the end face of the nozzle and the base metal is 7 mm, and the distance between the end of wire and the base metal is 5 mm. 

**Figure 2:**
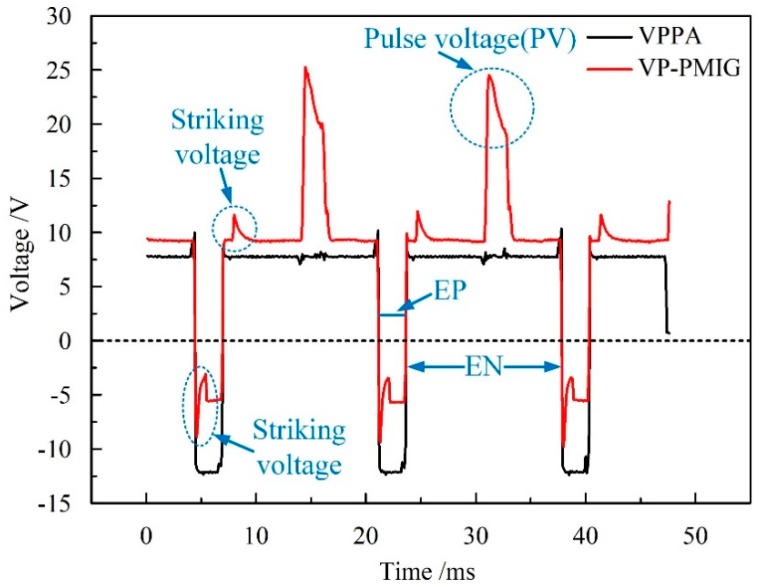
The VPPA and VP-PMIG arc voltages measured in a preliminary experiment.

To verify the coupling results of the VPPA and VP-PMIG arc, the VPPA and VP-PMIG arc voltages are measured in a preliminary experiment. Figure 2 shows the results of measuring the VPPA and VP-PMIG arc voltages, in which the voltage waveforms can be seen to be synchronous and of the same pulse width. In order to ensure the VP-PMIG arc can be ignited after arc polarity switching, a striking voltage needs to be present at the rising (falling) edge of the waveform. Furthermore, there is a pulse voltage in the electrode negative (EN) phase that is beneficial for droplet transfer. 

### 2.3. Experimental Design

To understand the characteristics of the hybrid arc and droplet transfer during one cycle, the main welding parameters were as follows. The durations of EN and electrode positive (EP) are 14.2 and 2.5 ms, respectively. The VPPA current in the EN and EP phases are 30 and 60 A, respectively. The VP-PMIG arc current in the EN and EP phases are 70 and 40 A. The value of the pulse voltage in the EN phase is 30 V and the duration of the pulse voltage is 1.05 ms. The plasma gas is pure Ar, its flow rate is 1.5 L/min. The shielding gas is pure Ar, its flow rate is 20 L/min. The duration of the striking current in the initial stage of the EN and EP phases is 1 ms. The characteristics of the hybrid arc and droplet transfer were observed by high-speed camera at 2000 fps, and the pixels of the image are 960 × 720.

In order to compare the influence of the VPPA and VP-PMIG arc currents on heat and mass transfer, only the case where both currents are varied is considered. Based on VPPA welding research, the effect of cathode cleaning is obvious when the duty cycle of the EN phase is 85%. Meanwhile, the welding quality is high when IVPPA-EP (EP phase VPPA current) is 30 A greater than IVPPA-EN (EN phase VPPA current). Hence, in this study, the duty cycle of the EN phase is 85%, and IVPPA-EP is 30 A greater than IVPPA-EN. For the convenience of this work, the difference between IVPPA-EN and IMIG-EN (EN phase VP-PMIG current) is variable. Through a large number of experiments, it is found that in order to ensure the welding quality, the VPPA current in EN phase is at least 30A. Meanwhile, in order to ensure the stable transfer of droplets, IMIG-EN needs to be between 70A and 100A. Furthermore, combining the above experiments, two groups of comparative experiments were performed. The key experimental parameters are shown in Table 3, where Vwire is the wire feed speed, and parameters not shown are held constant.

In order to analyze the characteristics of the hybrid arc, a high-speed video camera is used to dynamically observe the shape of the hybrid arc in parameter set 1 and carry out gray processing. The temperature distribution of the arc is indirectly represented by the gray distribution of the arc image [24]. Meanwhile, by analyzing the temperature distribution curve of the hybrid arc at different times, the law of temperature distribution of hybrid arc is studied.

According to the previous research, the volume of the droplet before falling can reflect the deposition rate [6]. In this paper, the two-dimensional cross-section of the droplet is obtained by a high-speed camera, and it can indirectly reflect the size of the droplet. Therefore, in order to study the influence of the VPPA and VP-PMIG arc on mass transfer, the growth rate of droplet cross-sectional area with increasing IVPPA-EN (parameter set 1–4) and IMIG-EN (parameter set 3, 5–7) was analyzed, respectively. 

Li et al. [4] found that decreasing the current flowing through the base metal can result in the melting area of the base metal reducing. In this paper, the area of the base metal melting area is used to indirectly reflect the heat transfer to the base metal. Therefore, the increased rate of the melt area of the base metal with increasing IVPPA-EN and IMIG-EN was analyzed, respectively, to study the influence of VPPA and VP-PMIG arc on heat transfer.

All the experiments in this paper were repeated three times. In order to obtain an objective and reliable experimental law, the experimental data are the average values of three experimental results.

## 3. Results and Discussion

### 3.1. Evolution of Hybrid Arc and Droplet Transfer

**Figure 3:**
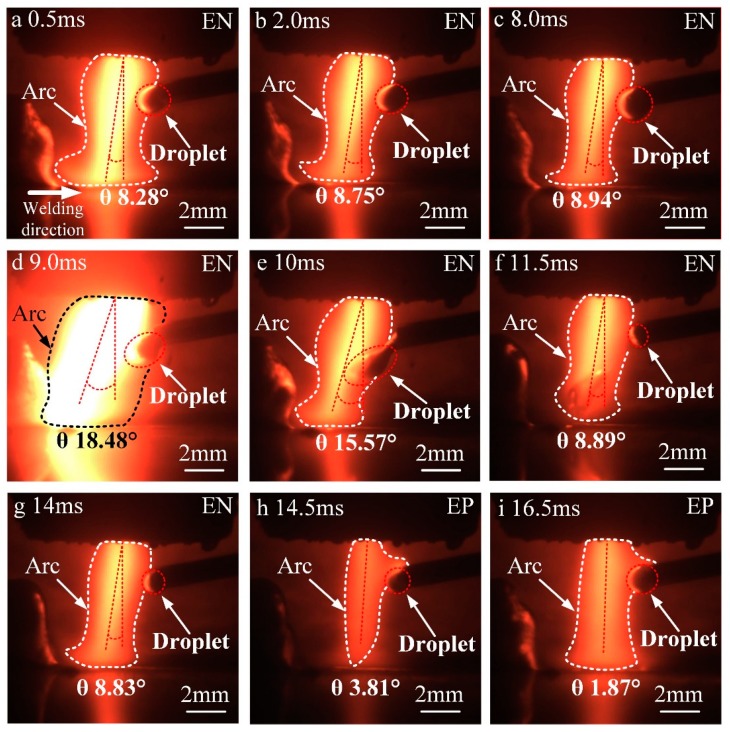
The evolution of the hybrid arc shape and droplet transfer at different times. (**a**) 0.5 ms, (**b**) 2.0 ms, (**c**) 8.0 ms, (**d**) 9.0 ms, (**e**) 10 ms, (**f**) 11.5 ms, (**g**) 14 ms, (**h**) 14.5 ms and (**i**) 16.5 ms.

Figure 3 shows the evolution of the hybrid arc and droplet transfer during one complete EN–EP phase. Compared with conventional VPPA, the width of the hybrid arc between the nozzle and welding wire is larger. In the EN phase, the shape of the hybrid arc looks like a gourd, while that of conventional VPPA looks like a bell. The VPPA current flows from the base metal to the tungsten electrode, and the VP-PMIG current flows from the wire to the tungsten electrode. Therefore, between the wire and the base metal, the arc shape is similar to that of conventional VPPA. Meanwhile, between the nozzle and the wire, the range of electronic motion is larger, resulting in a wider arc. The cathode spot is attracted to the superficial oxide of the wire because of its decreased work function. Hence, in the EP phase, the VP-PMIG arc at the end of the wire is more obvious, and the arc tends to climb up along the wire, as shown in Figure 3h,i.

In order to study the effect of droplet transfer on the shape of the hybrid arc, we define the angle between the arc axis and a surface normal extending from the base metal to the center of the tungsten tip as the arc deflection angle *θ* shown in Figure 3. From 0.5 to 8.0 ms, the value of *θ* increases gradually. The distance between the upper surface of the droplet and the central axis of the hybrid arc decreases with droplet growth. Therefore, the VP-PMIG arc gradually becomes closer to the VPPA. As both currents are flowing in the same direction they repel each other, the hybrid arc deflects along the negative *x*-axis. In Figure 3d, the value of *θ* surges to 18.48°, the maximum, and at that moment the VP-PMIG current reaches 380 A, the repulsive force between the currents increasing sharply. Therefore, the deflection angle of the hybrid arc increases greatly. In Figure 3e, the deflection angle of the hybrid arc decreases to 15.57° at 10 ms, and the main body of the droplet has entered the VPPA completely. The pulse current drops to zero and the VP-PMIG current returns to 70 A. In the next frame, the droplet falls off and the end of the welding wire melts again, as shown in Figure 3f. The VP-PMIG arc is separated from the VPPA, and the deflection angle of the hybrid arc decreases to the initial level. In the EP phase, the VP-PMIG arc climbs up along the wire, and the hybrid arc is deflected towards the *y*-axis due to the mutual attraction between currents.

**Figure 4:**
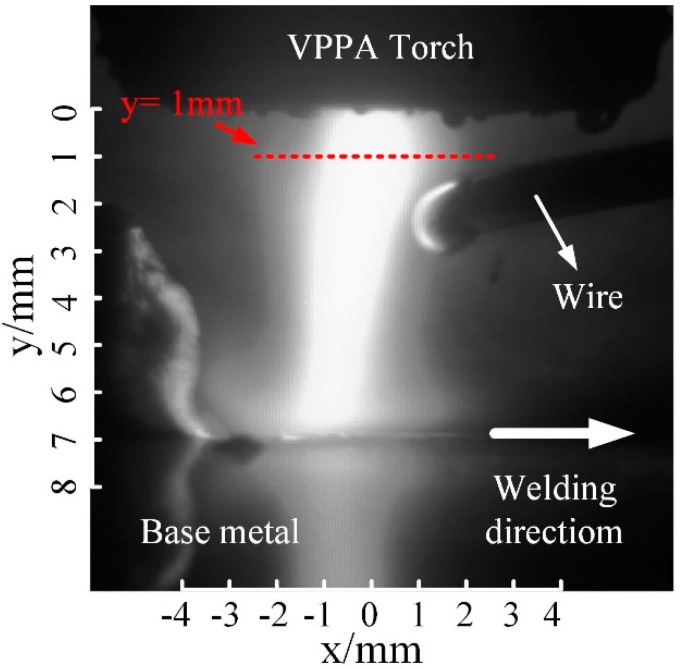
The grayscale image at 0.5 ms.

The brightness of the hybrid arc is determined by the current density, which is positively related to the temperature of the hybrid arc. Therefore, the brightness distribution of the hybrid arc can be considered indicative of its temperature distribution. Figure 4 shows a gray-scale image corresponding to the image captured by the high-speed camera at 0.5 ms. In Figure 4, a coordinate system is established in which the intersection of the tungsten axis and the nozzle end face is the origin. The width of the hybrid arc is largest near *y* = 1 mm, where the effect of arc coupling is optimal. Therefore, the temperature distribution along *y* = 1 mm can reflect the temperature distribution characteristics of the hybrid arc.

**Figure 5:**
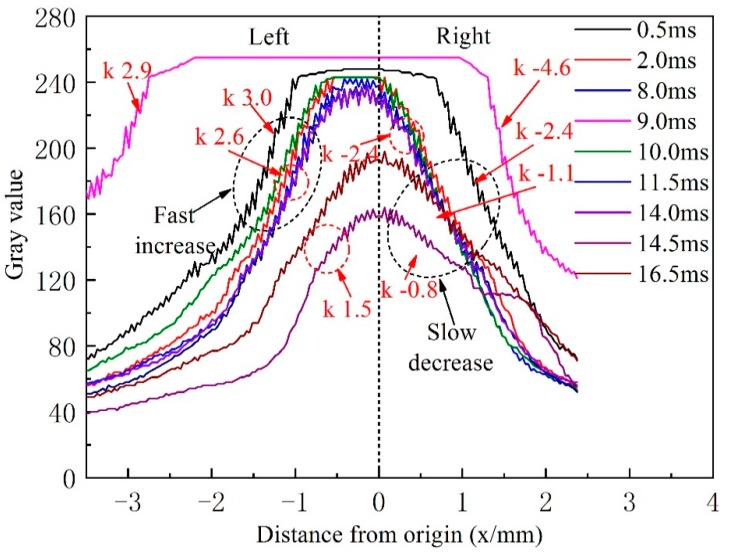
The distribution of gray values along the y = 1 mm of Figure 4 at different times, corresponding to Figure 3.

Figure 5 shows the distribution of gray values along the dashed red line, *y* = 1.0, at different times, corresponding to Figure 3. At 0.5 ms, the curve assumes a maximum value at *x* = −0.2 mm and changes little between −1.2 and 0.7 mm. This is because the current density of the hybrid arc increases due to the striking current, resulting in an increased temperature of the central area of the arc. When *x* < −1.2 mm, the curve slope is 3.0, whereas, when *x* > 0.7 mm, the curve slope is −2.4, thus, the temperature distribution along the horizontal line of the hybrid arc is asymmetric. On the right of the hybrid arc center, the temperature of the arc declines more slowly, because of the VP-PMIG arc and the associated increased current density. At 2.0 ms, the striking voltage drops to zero, resulting in the energy of VP-PMIG arc decreasing. Therefore, the high temperature area on the right side of the hybrid arc decreases. However, the rate of change of temperature on the right of the hybrid arc is still lower than that on the left. Because the voltage does not change, the curves at 8.0, 10.0, and 11.5 ms are similar to that at 2.0 ms. At 9.0 ms, however, the gray value reaches the maximum value between −2.2 and 1.0 mm and remains constant, because the pulse current causes the current density of the hybrid arc to increase rapidly. Furthermore, the temperature decline rate on the right of the hybrid arc is now greater than that on the left, and is attributed to the deflection of the hybrid arc.

The absolute values of the slope on the right side of the curves of the EP phase are much lower than those of the EN phase. Firstly, VPPA actively seeks oxide on the base metal in the EP phase so that the arc expands. Hence, the current density decreases, and the temperature of the arc center decreases. Secondly, the VP-PMIG arc climbs up along the wire in the EP stage, which leads to an increase of the area of the hybrid arc on the right of the distribution.

**Figure 6:**
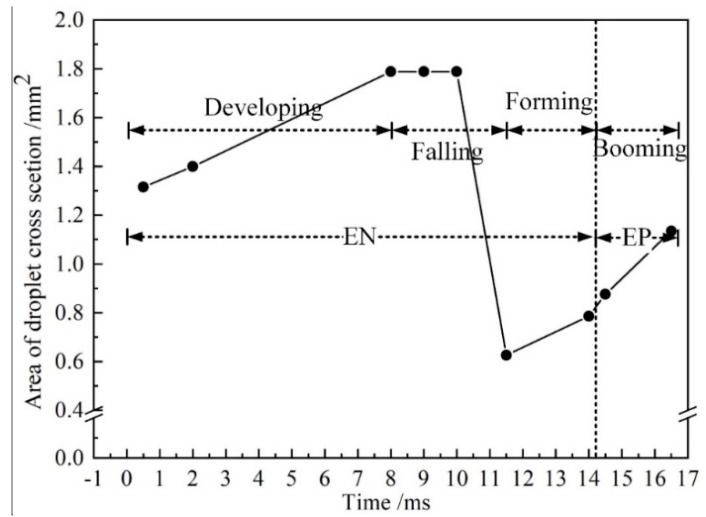
The evolution of the cross-sectional area of the droplet.

Figure 6 shows the evolution of the area of the droplet cross section with time. From 0.5 to 2.0 ms, the droplet cross-sectional area increases from 1.3 to 1.4 mm^2^, with a growth rate of 0.067 mm^2^/ms. From 2.0 to 8.0 ms, the droplet cross-sectional area increases from 1.4 to 1.8 mm^2^, with a rate of 0.065 mm^2^/ms. Thus, as the droplet grows steadily between 0 and 8.0 ms, this stage is referred to as the developing stage. From 8.0 to 11.5 ms, the droplet necks and falls off, which is referred to as the falling stage.

**Figure 7:**
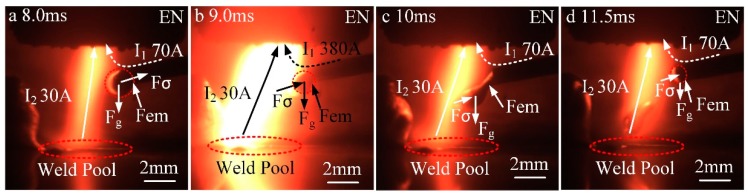
Analysis of forces acting on the anode droplet during the falling stage. (**a**) 8.0 ms, (**b**) 9.0 ms, (**c**) 10 ms and (**d**) 11.5 ms.

Figure 7 shows a force analysis on the anode droplet during the falling stage. The droplet is mainly affected by the gravity ($F_{g}$), the resultant force of surface tension ($F_{\sigma }$), and the electromagnetic force ($F_{em}$). The direction of $F_{g}$ is along the *y*-axis, which is conducive to drop fall off. The direction of $F_{em}$ is perpendicular to the axis of the wire, and it is beneficial to necking of the droplet. The direction of $F_{\sigma }$ is perpendicular to the root of the droplet, and it hinders the transfer of the droplet. The value of $F_{\sigma }$ is positively related to the cross-sectional area of the droplet root.

In Figure 7a, $F_{g}$ is in balance with $F_{\sigma }$, resulting in the droplet not being detached from the end of the wire. In Figure 7b, the VP-PMIG arc current ($I_{1}$) is 380 A at 9.0 ms, which is 5.4 times that at 8.0 ms. Due to the fact that $F_{em}$ is proportional to the square of the current, its value has been increased approximately 28-fold. This sudden increase of $F_{em}$ causes the droplet to neck near the end of the wire. Since the cross-sectional area of the droplet origin decreases, the surface tension decreases, and $F_{g}$ drives the droplet away from the end of the wire. In Figure 7c, necking of the droplet is intensified due to $F_{g}$, and as shown in Figure 7d, the droplet falls into the pool, and the wire starts to melt to form a new droplet.

From 11.5 to 14 ms, the cross-sectional area of the droplet increases from 0.62 to 0.78 mm^2^, and the growth rate is 0.064 mm^2^/ms. During this period, the amount of molten metal at the end of the wire is small and the droplet grows steadily. Therefore, the stage between 11.5 and 14 ms is defined as the forming stage. From 14 to 14.5 ms, the cross-sectional area of the droplet increases from 0.79 to 0.88 mm^2^, and the change rate is 0.18 mm^2^/ms, which is 2.8 times of that in the falling stage. During this period, Ohmic heats at the end of wire, due to the striking voltage. Additionally, the melting point of the wire is low (melting range 574–632 °C), so that the cathode wire mainly emits electrons through field emission. Due to the fact that the cathode voltage is higher than the anode voltage, the power is increased. Therefore, the rate of wire melting is higher in the EP phase. Meanwhile, from 14.5 to 16.5 ms, the cross-sectional area of the droplet increases from 0.88 to 1.14 mm^2^ with a growth rate of 0.13 mm^2^/ms. The growth rate of the cross-sectional area of the droplet in EP stage is much higher than that in the development and forming stages, and thus, this stage is defined as the booming stage.

Droplet growth mainly occurs in the forming, booming, and developing stages, with incremental increases in the cross-sectional area of 0.24, 0.26, and 0.65 mm^2^ respectively. In the EN stage, the incremental increase of the cross-sectional area of the droplet accounts for 77.53% of the total increment. Therefore, it is considered that the currents in the EN stage dominate mass transfer.

### 3.2. Influence of VPPA and VP-PMIG Arc Current on Mass Transfer

**Figure 8:**
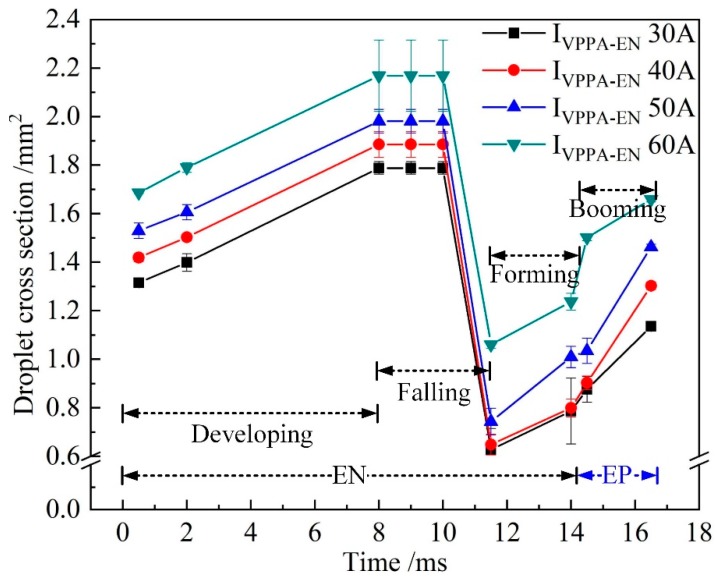
The evolution of droplet cross section when *I*_VPPA-EN_ is varied and *I*_MIG-EN_ is fixed at 70 A.

Figure 8 shows the evolution of the droplet cross-sectional area when *I*_VPPA-EN_ is varied and *I*_MIG-EN_ is fixed at 70 A. With increasing *I*_VPPA-EN_, the droplet cross-sectional area of each stage increases, indicating that the VPPA current has a certain effect on wire melting. At 8.0 ms, the cross section area of the droplet can reflect the size of droplet before falling off. At 11.5 ms, the cross section area of the droplet can reflect the size of the droplet after falling off. Therefore, the mass transfer can be indirectly reflected by the difference of the droplet cross section area between 8 and 11.5ms. 

**Figure 9:**
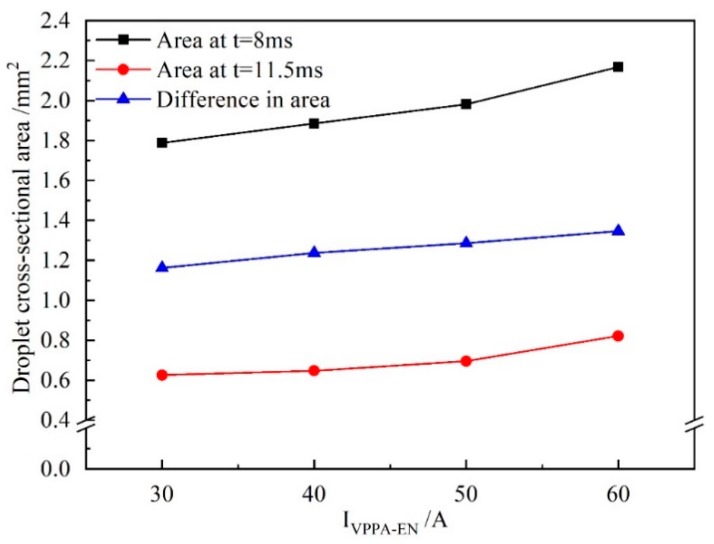
The difference in droplet cross-sectional area between 8 and 11.5 ms for varied *I*_VPPA-EN_ and *I*_MIG-EN_ fixed at 70 A.

Figure 9 shows the difference in the droplet cross-sectional area between 8 and 11.5 ms when *I*_VPPA-EN_ is varied. When *I*_VPPA-EN_ is 30 A, the cross-sectional area of the droplet at 8 and 11.5 ms are 1.79 and 0.63 mm^2^, respectively, and the difference is 1.16 mm^2^. When *I*_VPPA-EN_ is 60 A, the difference increases to 1.35 mm^2^. Therefore, the rate of change of the droplet cross-sectional area with increasing *I*_VPPA-EN_ is 0.006 mm^2^/A.

**Figure 10:**
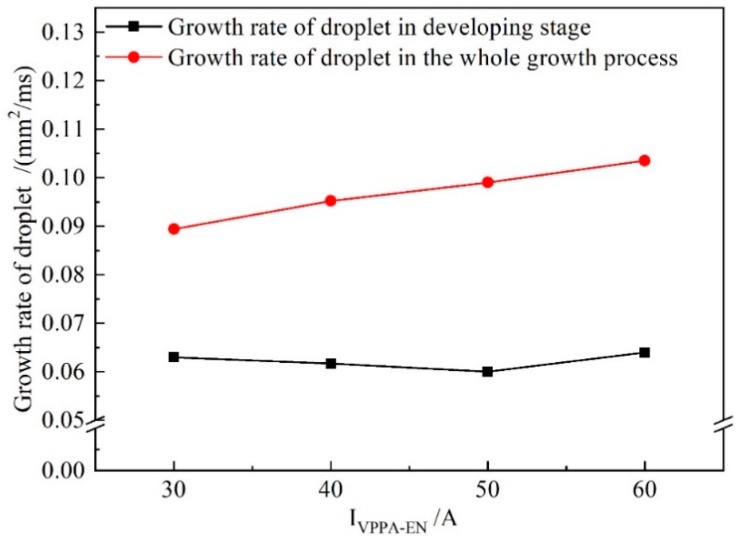
The growth rate of droplet cross-sectional area in the developing stage and during the whole growth process when *I*_VPPA-EN_ is increased and *I*_MIG-EN_ is fixed at 70 A.

Figure 10 shows the growth rate of the droplet cross-sectional area for increasing *I*_VPPA-EN_ during the developing stage and the whole growth process (forming, booming and developing). When *I*_VPPA_ increases from 30 to 60 A, the growth rate of the droplet cross-sectional area in the developing stage is almost unchanged. Meanwhile, the growth rate of the droplet in the whole growth process increases with increasing VPPA current. Thus, the VPPA current has little effect on the growth rate of the droplet during the development stage. Due to the fact that the welding parameters of the forming stage are the same as those of the developing stage, the VPPA current also has little effect on the growth rate of the forming stage. Therefore, when the VPPA current increases, the effect of the VPPA on the mass transfer mainly occurs in the EP stage. It is considered that the heat absorbed by the wire to melt comes from two sources. Heating from the VP-PMIG arc established between the tungsten electrode and wire will play an important role in melting the wire. Furthermore, energy from the VPPA will heat the wire end when it enters into the VPPA. *I*_VPPA-EP_ is 30 A higher than *I*_VPPA-EN_, so the effect of VPPA on wire melting in the EP phase is more obvious.

**Figure 11:**
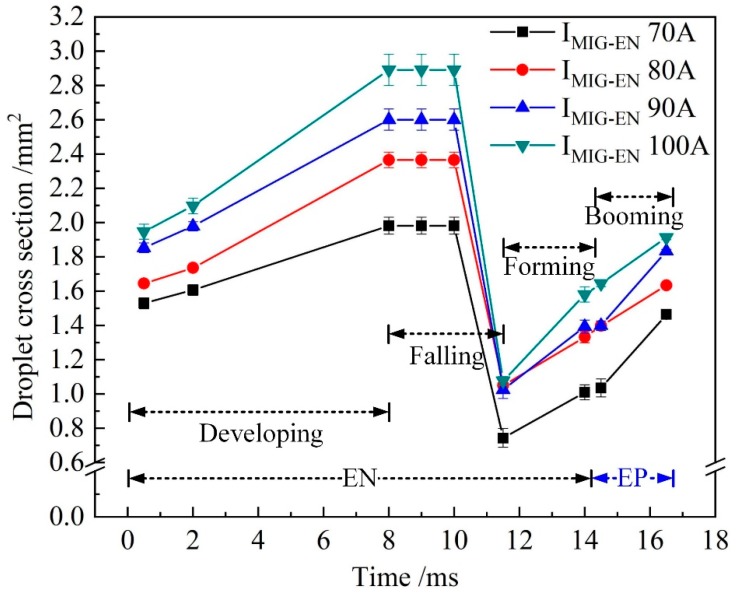
The evolution of droplet cross-sectional area when *I*_MIG-EN_ is increased and *I*_VPPA-EN_ is maintained at 50 A.

Figure 11 shows the evolution of the droplet cross-sectional area when *I*_MIG-EN_ is changed and *I*_VPPA-EN_ is maintained at 50 A. With the increase of *I*_MIG-EN_, the growth rate of the droplet cross-sectional area during the developing stage increases, and the increment of the droplet cross-sectional area at 8.0 ms also increases. This shows that increasing the VP-PMIG current is more conducive to wire melting than increasing the VPPA current. 

**Figure 12:**
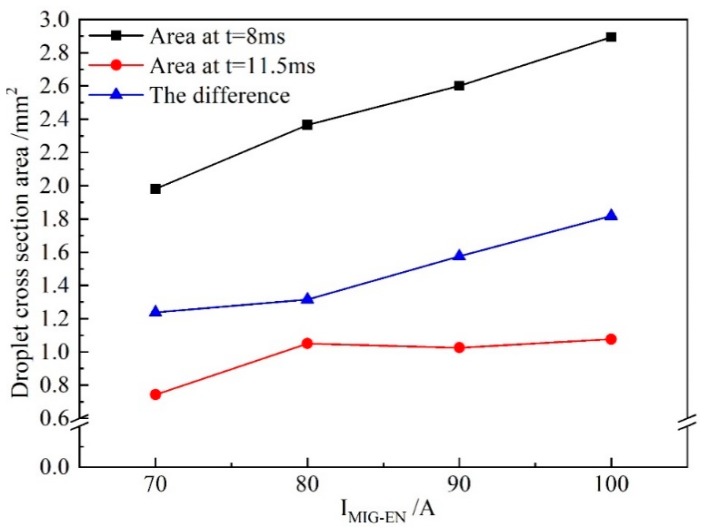
The difference between droplet cross-sectional area when *I*_VPPA_ is changed and *I*_MIG-EN_ is maintained at 70 A, at 8 and 11.5 ms.

Figure 12 shows the difference in the droplet cross-sectional area at 8 and 11.5 ms with *I*_MIG-EN_ changing. When *I*_MIG-EN_ is 70 A, the cross-sectional areas of the droplet at 8 and 11.5 ms are 1.98 and 0.74 mm^2^ respectively, and the difference is 1.24 mm^2^. When *I*_MIG-EN_ is 100 A, the cross-sectional areas at 8 and 11.5 ms are 2.9 and 1.1 mm^2^ respectively, and the difference is 1.8 mm^2^. Therefore, the rate of change of the droplet cross-sectional area with *I*_MIG-EN_ increasing is 0.019 mm^2^/A, which is 3.17 times that with *I*_VPPA-EN_ increasing. This indicates that the VPPA and VP-PMIG arc currents influence the mass transfer by 24% and 76%, respectively.

**Figure 13:**
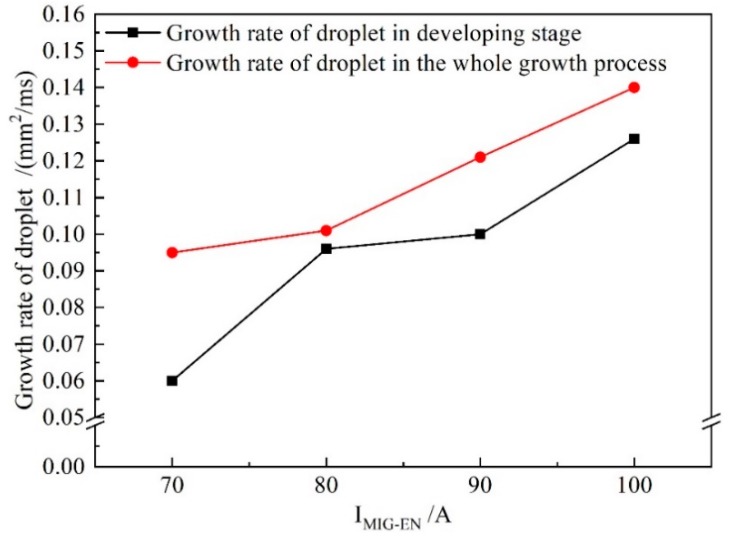
The growth rate of droplet cross-sectional area in the developing stage and the whole growth process when *I*_MIG-EN_ is increased and *I*_VPPA-EN_ is maintained at 50 A.

Figure 13 shows the growth rate of the droplet cross-sectional area with *I*_MIG-EN_ increasing in the developing stage and the whole growth process. In Figure 13, the growth rate of the droplet in the development stage obviously increases with increasing current. Meanwhile, when *I*_MIG-EN_ exceeds 80 A, the growth rate of the droplet in the developing stage reaches 82% of that in the whole process. Therefore, the effect of the VP-PMIG arc current on the melting wire in the EN phase is more obvious.

### 3.3. Influence of VPPA and VP-PMIG Arc Current on Heat Transfer 

**Figure 14:**
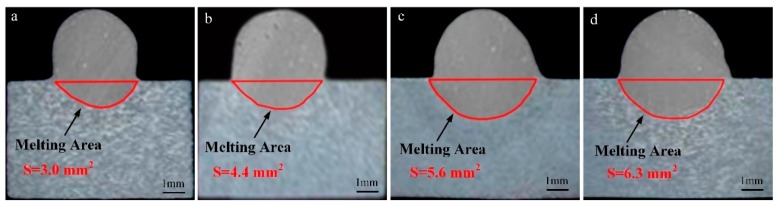
Weld shape and melting area of base metal when *I*_VPPA-EN_ is varied and *I*_MIG-EN_ is maintained at 70 A, for *I*_VPPA-EN_ of (**a**) 30 A, (**b**) 40 A, (**c**) 50 A, and (**d**) 60 A.

**Figure 15:**
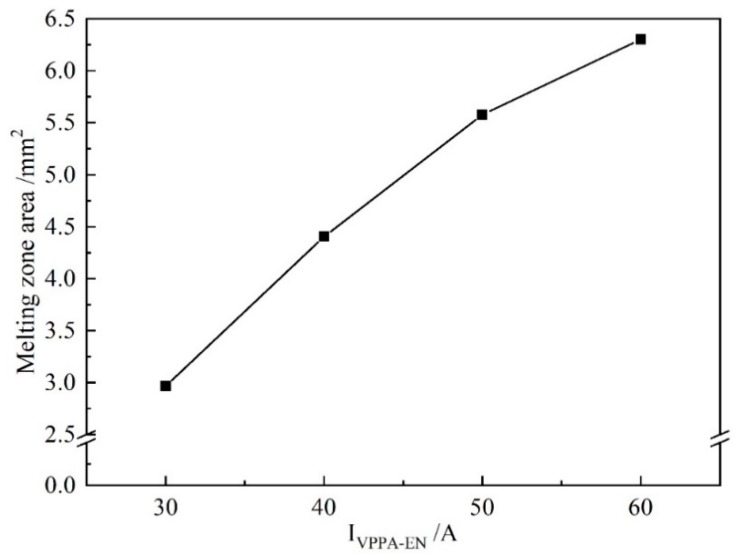
The melting area of the base metal for increasing *I*_VPPA-EN_.

Figure 14 shows the weld shape and melting area of the base metal when *I*_VPPA-EN_ is varied and *I*_MIG-EN_ is maintained at 70 A. In Figure 14a, the melting area is 3.0 mm^2^. In Figure 14d, the melting area is 6.3 mm^2^. When *I*_VPPA-EN_ is increased from 30 to 60 A, the melting area of the base metal increases by 3.3 mm^2^, and the average increase rate is 0.11 mm^2^/A. Figure 15 directly shows the trend of the change of melting area with *I*_VPPA-EN_.

In Figure 15, with increasing *I*_VPPA-EN_, the rate of increase of the melting area of the base metal decreases. When *I*_VPPA-EN_ increases from 30 to 40 A, the melting area increases from 3.0 to 4.4 mm^2^. The melting area of the base metal increases with *I*_VPPA-EN_ at a rate of 0.14 mm^2^/A. Then, when *I*_VPPA-EN_ increases from 40 to 50 A, the melting area increases from 4.4 to 5.6 mm^2^, and the corresponding increase rate is 0.12 mm^2^/A. When *I*_VPPA-EN_ increases from 50 to 60 A, the melting area increases by 0.7 mm^2^, and the corresponding growth rate is 0.07 mm^2^/A. It is found that the maximum value of the increase rate is twice the minimum value. On the one hand, the increase of *I*_VPPA-EN_ will increase the heat input to the base metal, so the melting area will increase to a certain extent. On the other hand, the thermal conductivity of the aluminum alloy is very high, thus with increased heat input, heat conduction loss also increases correspondingly.

**Figure 16:**
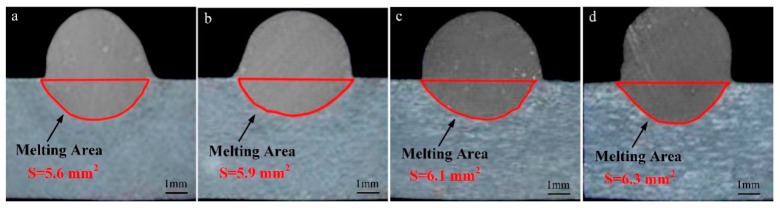
Weld shape and melting area of the base metal when *I*_MIG-EN_ is varied and *I*_VPPA-EN_ = 50 A for *I*_MIG-EN_ of (**a**) 70 A, (**b**) 80 A, (**c**) 90 A, and (**d**) 100 A.

**Figure 17:**
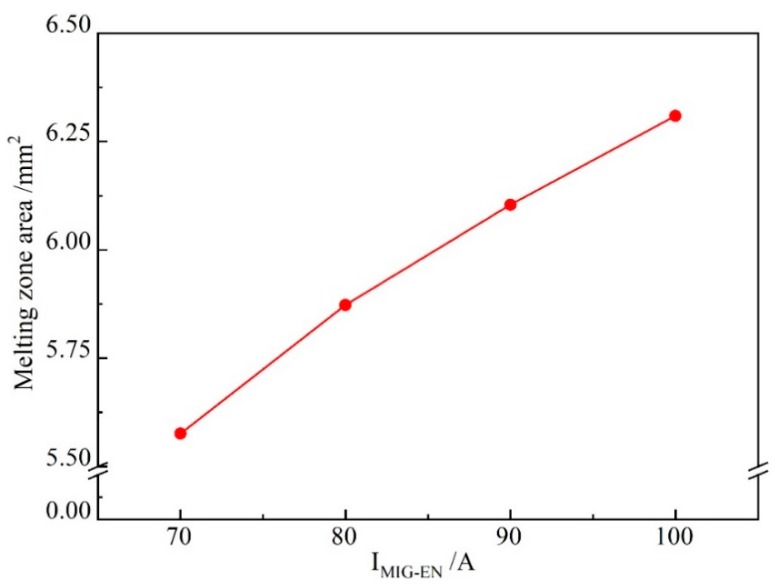
The melting area of the base metal for increasing *I*_MIG-EN_.

Figure 16 shows the weld shape and melting area of the base metal when *I*_MIG-EN_ is varied and *I*_VPPA-EN_ = 50 A. In Figure 16a, the melting area is 5.6 mm^2^. In Figure 16d, the melting area is 6.3 mm^2^. When the *I*_MIG-EN_ increases from 70 to 100 A, the melting area of the base metal increases by 0.7 mm^2^. Figure 17 directly shows the trend of change of the melting area with *I*_MIG-EN_.

In Figure 17, when *I*_MIG-EN_ increases from 70 to 80 A, the melting area increases from 5.6 to 5.9 mm^2^. The melting area of the base metal increases with *I*_MIG-EN_ at a rate of 0.03 mm^2^/A. Then, when *I*_MIG-EN_ increases from 80 to 90 A, the melting area increases from 5.9 to 6.4 mm^2^, and the corresponding increase rate is 0.05 mm^2^/A. When *I*_MIG-EN_ increases from 90 to 100 A, the melting area increases by 0.2 mm^2^, and the corresponding increase rate is 0.02 mm^2^/A. When *I*_MIG-EN_ increases from 70 to 100 A, the average increase rate is 0.023 mm^2^/A. Therefore, on average, the contribution of *I*_VPPA-EN_ to the heat transfer of base metal melting is about 4.5 times that of *I*_MIG-EN_. This indicates that the VPPA and VP-PMIG currents control the heat transfer by 82% and 18%, respectively.

It is considered that the VPPA is established between the tungsten electrode and the base metal. The heat sources of the VPPA to the base metal include: heat conduction of the VPPA to the base metal and Ohmic heating generated by the VPPA current flowing through the base metal. In addition, in the EN stage, the base metal is heated by electron condensation. In the EP stage, the base metal is heated by ion recombination. However, the VP-PMIG arc is between the tungsten electrode and wire. The thermal effect of the VP-PMIG arc on the base metal is limited to heat conduction. Therefore, VPPA plays a leading role in the heat transfer of the base metal.

## 4. Conclusions

This work mainly investigates the characteristics of arc evolution and droplet transfer in arcing-wire VPPA. Using a comprehensive experimental measurement of the droplet cross section area and the melting area of the base metal, the decoupling degree of heat and mass transfer were obtained. The specific conclusions of the study are as follows:

(1) The shape of the hybrid arc is gourd-like. Influenced by the VP-PMIG arc, the hybrid arc will deflect and the temperature distribution is asymmetric. Droplet necking occurs in the pulse stage.
(2) The droplet transfer can be divided into four stages of developing, falling, forming, and booming. The highest droplet growth rate occurs in the booming stage. The increment of the droplet in the developing and forming stages accounts for 77.53% of the total increment.
(3) The droplet cross-sectional area changes more obviously by adjusting the VP-PMIG current than the VPPA current. The VPPA and VP-PMIG currents control the mass transfer by 24% and 76%, respectively. When the VPPA current alone is increased, the effect of the VPPA on the mass transfer mainly occurs in the EP stage.
(4) The VPPA current is dominant on the melting area of the base metal, and heat transfer to the base metal is mainly controlled by the VPPA. The VPPA and VP-PMIG currents control the heat transfer by 82% and 18%, respectively.

## Figures and Tables

**Table 1:** Chemical composition of 5A06 aluminum alloy (%).

Mg	Mn	Si	Fe	Zn	Cu	Ti	Be	Al
5.8–6.8	0.5–0.8	≤0.4	≤0.4	≤0.2	≤0.1	0.02–0.1	0.0001–0.005	balance

**Table 2:** Chemical composition of ER4043 (%).

Si	Cu	Mg	Fe	Al
5	≤0.05	≤0.1	≤0.04	balance

**Table 3:** Experimental parameters.

Parameter Set	*I*_VPPA-EN_ (A)	*I*_VPPA-EP_ (A)	*I*_MIG-EN_ (A)	*V*_wire_ (m/min)
1	30	60	70	5.3
2	40	70	70	5.4
3	50	80	70	5.5
4	60	90	70	5.7
5	50	80	80	5.8
6	50	80	90	6.1
7	50	80	100	6.4
